# Possible Role of Endothelial-Derived Cellular and Exosomal-miRNAs in Lipid-Mediated Diabetic Retinopathy: Microarray Studies

**DOI:** 10.3390/cells13221886

**Published:** 2024-11-15

**Authors:** Khaled Elmasry, Samar Habib, Inas Helwa, Mariam Lotfy Khaled, Ahmed S. Ibrahim, Amany Tawfik, Mohamed Al-Shabrawey

**Affiliations:** 1 Department of Oral Biology and Diagnostic Sciences, The Dental College of Georgia, Augusta University, Augusta, GA 30912, USA; dr_samarhabib@mans.edu.eg; 2 DCG Center for Excellence in Research, Scholarship, and Innovation (CERSI), Augusta University, Augusta, GA 30912, USA; 3 Department of Cellular Biology and Anatomy, The Medical College of Georgia, Augusta University, Augusta, GA 30912, USA; inas.helwa@miuegypt.edu.eg (I.H.); elmsha-pharma@hotmail.com (M.L.K.); 4 Department of Human Anatomy and Embryology, Faculty of Medicine, Mansoura University, Mansoura 35516, Egypt; 5 Department of Medical Parasitology, Faculty of Medicine, Mansoura University, Mansoura 35516, Egypt; 6 Department of Histopathology, Faculty of Oral and Dental Medicine, Misr International University, Cairo 19648, Egypt; 7 Department of Biochemistry, Faculty of Pharmacy, Cairo University, Cairo 11562, Egypt; 8 Department of Ophthalmology, Visual, and Anatomical Sciences, School of Medicine, Wayne State University, Detroit, MI 48201, USA; gt3759@wayne.edu; 9 Department of Biochemistry, Faculty of Pharmacy, Mansoura University, Mansoura 35516, Egypt; 10 Department of Pharmacology, School of Medicine, Wayne State University, Detroit, MI 48201, USA; 11 Eye Research Institute, Oakland University, Rochester, MI 48309, USA; amtawfik@oakland.edu; 12 Eye Research Center (OUWB)/ERC, William Beaumont School of Medicine, Rochester, MI 48073, USA; 13 Department of Foundational Medical Studies and Eye Research Center, Oakland University William Beaumont School of Medicine, Rochester, MI 48309, USA

**Keywords:** diabetic retinopathy, miRNAs, exosomes, extracellular vesicles, retinal endothelial cells, lipoxygenase

## Abstract

Diabetic retinopathy (DR) is a salient cause of blindness worldwide. There is still an immense need to understand the pathophysiology of DR to discover better diagnostic and therapeutic modalities. Human retinal endothelial cells (HRECs) were treated with 15-HETE or D-glucose, then miRNAs were isolated, and a microarray was performed. MirWALK 2 and Ingenuity Pathway Analysis (IPA) were used to analyze the microarray results. Exosomal miRNAs from 15-HETE-treated HRECs were isolated, microarrayed, and then imported into IPA for further analysis. The microarray results showed that 15-HETE downregulated 343 miRNAs and upregulated 297 miRNAs in HRECs. High glucose treatment induced a differential expression of HREC-miRNAs where 185 miRNAs were downregulated and 244 were upregulated. Comparing the impact of 15-HETE versus DG or diabetic mouse retina elaborated commonly changing miRNAs. Pathway and target analysis for miRNAs changed in 15-HETE-treated HRECs revealed multiple targets and pathways that may be involved in 15-HETE-induced retinal endothelial dysfunction. The HREC-exosomal miRNAs were differentially expressed after 15-HETE treatment, with 34 miRNAs downregulated and 45 miRNAs upregulated, impacting different cellular pathways. Here, we show that 15-HETE induces various changes in the cellular and exosomal miRNA profile of HRECs, highlighting the importance of targeting the 12/15 lipoxygenase pathway in DR.

## 1. Introduction

Diabetic retinopathy (DR) is a leading cause of vision loss in the middle-aged working group [1]. Diabetes-induced retinal inflammation causes disrupted retinal blood barriers followed by diabetic macular edema and ends with abnormal retinal neovascularization [2]. Currently, available therapies for DR are mostly invasive strategies that treat the late stages of the disease. There is an immense need to understand the pathophysiology of diabetic retinopathy and to discover not only new markers but also novel therapies that can target the early stages of the disease. The retina is a lipid-rich tissue. Dysregulated lipid signaling was reported to be implicated in the inflammation process that happens early during diabetes [3,4,5]. Eicosanoids are the larger group of lipids that are generated via the action of enzymes such as lipoxygenases, cytochrome-P450, and cyclooxygenases on the arachidonic acid released from the cell membrane by phospholipase enzyme (cPLA) [6]. These enzymes are targets for microRNAs (miRNAs). miRNAs were reported to have a role in the regulation of the function of these salient lipid-generating enzymes [7,8].

It was reported that enzymes such as lipoxygenase (LO) under diabetic conditions can generate pro-inflammatory lipid mediators inside the retina [9]. 12- and 15-Hydroxyecosatetraneoic acids (12- and 15-HETEs) are among those pro-inflammatory mediators that were reported to induce retinal microvascular dysfunction via multiple mechanisms such as inducing retinal endoplasmic reticulum (ER) stress and oxidative stress [10].

Previous work by our group has established a significant role of 12/15 LO in the development of DR. Significant increases in the levels of HETEs were detected in the vitreous humor of DR patients and the retinas of diabetic animal models [9]. Moreover, a disrupted blood–retinal barrier and increased inflammatory mediators were reported in wild-type mouse retinas after intravitreal injection of HETEs [4].

Dissecting the underlying mechanism of LO-induced retinal microvascular dysfunction in DR elaborated the possible role of NADPH oxidase-induced reactive oxygen species (ROS) generation, ER stress, and intracellular calcium homeostasis disruption [10].

Since the early work of Fire and Mello that described small pieces of RNA capable of destroying larger pieces of mRNAs interfering with their gene functions, RNA interference and its role in gene regulation gained much attention as a novel way of gene regulation [11]. miRNAs are small-sized endogenous non-coding RNAs that play an essential role in regulating gene expression via silencing their target RNAs. Several studies have been conducted to study the role of miRNAs in gene expression across different tissues. In the field of DR, miRNAs were described to play an essential role in the pathogenesis of diabetic retinopathy [12,13,14,15]. Moreover, several miRNAs are proposed to be novel biomarkers and even novel therapeutics for DR. Differential expression of miRNAs under diabetic conditions with subsequent change in their downstream targets underline their importance as possible new avenues for novel discoveries in the field of DR. Screening studies such as microarrays represented a potential way to uncover multiple novel biomarkers and therapies for DR.

Extracellular vesicles (EVs) include exosomes, microvesicles (MVs), and apoptotic bodies. Exosomes are nano-sized vesicles ranging from 30 to 150 nm in diameter. They represent a critical intercellular communication method. Exosomal cargo includes miRNAs, mRNAs, proteins, and lipids, which are transported to distant sites and released into different body fluids [16,17,18,19]. The retina contains millions of cells talking to each other via released exosomes [20]. Retinal endothelial cells can use their released exosomes to communicate with other retinal cells, such as Müller glial cells, photoreceptors, or retina pigment epithelium (RPE) [21]. Endothelial-derived exosomal miRNAs can participate in gene regulation in other retinal cells after their intake by the recipient cells. They may represent an early regulatory message sent from the endothelial cells exposed to high blood glucose levels during diabetes. Studying the changes in the exosomal miRNA profile can elucidate novel biomarkers and therapeutic targets for DR [22].

The current study aimed to investigate changes in the miRNA profile of retinal endothelial cells exposed to the pro-inflammatory lipid product, 15-HETE. Furthermore, we examined miRNA profile changes in retinal endothelial cells under high glucose conditions and compared these with the lipid-induced miRNA changes. Our analysis revealed similarities in miRNA profile changes induced by 15-HETE and high glucose. Moreover, we identified miRNAs that were commonly changed in both endothelial cells challenged with 15-HETE and in the retina of diabetic mice. These similarities suggest that the pro-inflammatory effect of 15-HETE may mimic, in part, the impact of high glucose levels on retinal endothelial cells during DR. Furthermore, we explored changes in the miRNA profile of endothelial cell-derived exosomes under the influence of 15-HETE. The differential expression of these endothelial-derived cellular and exosomal miRNAs could serve as new diagnostic and therapeutic targets. 

## 2. Materials and Methods

### 2.1. Cell Culture

Human primary retinal endothelial cells (HRECs) were purchased from Cell Systems Cooperation (Kirkland, WA, USA) and were cultured in plates coated with gelatin in EBM2 Medium (Catalog #190860, Lonza, Walkersville, MD, USA) supplemented with 5% fetal bovine serum (FBS) and 1% penicillin–streptomycin (PS, Catalog # 30-004-CI (Corning, Inc., Corning, NY, USA). When the cells reached 80–90% confluency, cells were washed using phosphate-buffered saline (PBS), followed by adding FBS-free media overnight. Next, cells were treated with 15-HETE (0.1 µM, Cayman Chemical, Ann Arbor, MI, USA) or vehicle (ethanol) for 24 h. Moreover, HRECs were treated with either normal glucose (5 mM D-glucose), osmotic control (5 mM D-glucose+25 mM L-glucose), or high glucose (30 mM-D-glucose) for 5 days.

### 2.2. RNA Isolation, Preparation and Analysis

Cells were collected by scrapping, and total RNA was extracted using miRNeasy Kit for miRNA purification (Qiagen, Germantown, MD, USA). RNA purity and concentration were evaluated by spectrophotometry using a NanoDrop ND-1000 (ThermoFisher, Waltham, MA, USA). Quality and the related size of total and small RNA were assessed by the Agilent 2200 TapeStation (Agilent Technologies, Santa Clara, CA, USA). 

### 2.3. Microarray Analysis

A total of 250 ng of total RNA was labeled with biotin using the FlashTag Biotin HSR RNA Labeling Kit (Affymetrix, Santa Clara, CA, USA) according to the manufacturer’s procedure. The labeled samples were then hybridized to the GeneChip miRNA 4.0 array (Affymetrix), which contains 2578 and 2025 human mature and premature miRNAs, respectively. Array hybridization, washing, and scanning of the arrays were carried out according to Affymetrix’s recommendations. Data were obtained in the form of a CEL file. The CEL files were imported into Partek Genomic Suites version 6.6 (Partek, St. Louis, MO, USA) using a standard import tool with RMA normalization. Principal component analysis (PCA) was performed to visualize the partition among the groups and identify the major sources of variation within the experiment. The differential expressions were calculated using ANOVA of the Partek Package and filtered with a *p*-value cutoff of 0.05 and a fold-change cutoff shown in each table to screen highly significant miRNAs. The significant miRNA lists were used to generate hierarchical clustering plots. 

### 2.4. Bioinformatics Analysis of the Data

To search for the predicted target genes of miRNAs and associated pathways, two strategies were used: (a) mirWALK 2 analysis [23] and (b) importing the miRNA lists into Ingenuity Pathway Analysis (Qiagen) and analyzing them on MicroRNA Target Filter and Core Analysis. 

### 2.5. Exosomes Isolation from HREC Culture Media 

HRECs were maintained in 5% FBS-supplemented media, as mentioned previously, then washed with PBS and maintained in FBS-free media with either 15-HETE (0.1 µM) or vehicle for 24 h. Then, FBS-free conditioned media were collected and then centrifuged for 30 min at 2000× *g* to remove any cells or debris. Then, the exosomes were isolated using Invitrogen Total Exosome Isolation Reagent (from cell culture media) (Catalog#: 4478359) according to the manufacturer’s instructions. Briefly, 0.5 volumes of the exosome isolation reagent were added to the media and then mixed by vortexing. After being kept overnight at 4 °C, the mixture was centrifuged at 10,000× *g* for an hour at 4 °C, supernatants were removed, and pellets were suspended in PBS and stored at −80 °C for further analysis.

### 2.6. Zeta View Nanoparticle Tracking Analysis (NTA), Transmission Electron Microscopy (TEM), CD-63 Immunogold Labeling of Exosomes 

**Figure 1:**
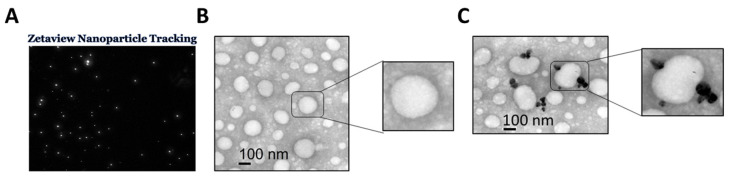
Characterization of exosomes using Zetaview nanoparticle tracking (**A**), transmission electron microscopy (TEM) (**B**), and CD-63 immunogold labeling (**C**).

The size and concentration of the isolated exosomes were measured using NTA, which was carried out utilizing the ZetaView PMX 110 (Particle Metrix, Meerbusch, Germany) and its related software (ZetaView 8.02.28) [24]. Each sample was measured at 11 different positions, and then the size and concentration of each sample were quantified. ZetaView 8.02.28 software was used to analyze measurement data from the ZetaView. Imaging of exosomes was performed using the standard protocol at the histology core of the Cell Biology and Anatomy department at MCG, Augusta University, Augusta, GA [25,26]. Briefly, for TEM and CD-63 immunogold labeling of exosomes, exosome samples were fixed in 4% paraformaldehyde overnight. The suspended exosome preparation was applied to a carbon-Formvar-coated 200 mesh nickel grid (Electron Microscopy Sciences, Ft. Washington, PA, USA) and allowed to stand for 30 min. Grids were floated exosome-side down onto a 20 µL drop of 1 M Ammonium Chloride for 30 min. Next, grids were floated on drops of Aurion Blocking buffer (Electron Microscopy Sciences) for one hour, then rinsed five times each with PBS. Grids were incubated on drops of primary antibody diluted 1:100 in blocking buffer for one hour, then washed five times in PBS. Following this, grids were floated on drops of species-specific Aurion Ultra Small gold (Electron Microscopy Sciences) diluted 1:200 in a blocking buffer for one hour before being enhanced for 10 min in HQ Silver (Nanoprobes, Inc. Yaphank, NY, USA) and rinsed in ice-cold de-ionized H_2_O. Then, grids were negatively stained in 2% aqueous Uranyl Acetate and wicked dry. Finally, Grids were examined using a JEM 1400 Flash transmission electron microscope (JEOL USA Inc., Peabody, MA, USA) at 110 kV and imaged with a Gatan One View Digital Camera (Gatan Inc., Pleasanton, CA, USA) (Figure 1).

### 2.7. Exosomal RNA Isolation and Measurement 

RNA isolation was carried out using the total exosome RNA & Protein Isolation Kit (catalog # 4478545; Invitrogen, Waltham, MA, USA) according to the manufacturer’s instructions. A final volume of 30 μL RNA solution was collected from each. Agilent 2100 Bioanalyzer (Santa Clara, CA, USA) was used for measuring RNA quality and concentration at the Integrated Genomics Core of Georgia Cancer Center at Augusta University.

### 2.8. Statistical Analysis

The differential expressions were calculated using ANOVA of the Partek Package, Partek Genomics Suite version 6.6 (Partek Incorporated, St. Louis, MO, USA). The fold change was calculated using the least squares means of the groups. Data are considered statistically significant when *p*-value < 0.05. 

## 3. Results

### 3.1. Differential Expression Profile of miRNAs in HRECs Treated with 15-HETE

**Figure 2:**
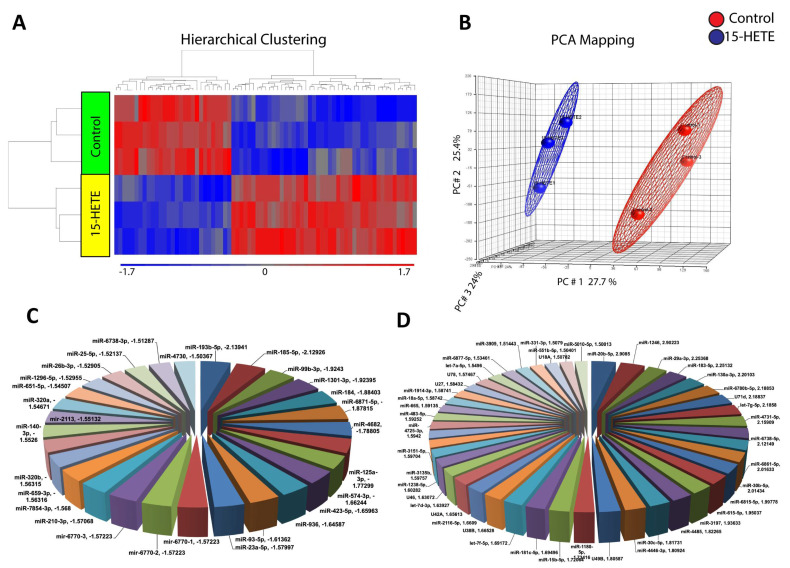
Impact of 15-HETE on miRNAs profile of HRECs. Heat map demonstrating differential expression of miRNAs in control versus 15-HETE-treated HRECs. The blue color denotes downregulated miRNAs, and the red color signifies upregulated miRNAs (**A**). PCA mapping shows a distinction between different groups with less variation within the experiment (**B**). Pie chart demonstrating downregulated (**C**) and upregulated (**D**) miRNAs in HRECs in response to 15-HETE.

To investigate the impact of the pro-inflammatory lipid metabolite 15-HETE on the miRNA profile of HRECs, we treated HRECs with 15-HETE for 24 h, followed by miRNA isolation and microarray analysis. The microarray results revealed significant changes in miRNA expression compared to vehicle-treated HRECs (Figure 2). Specifically, 343 miRNAs were significantly downregulated, while 297 miRNAs were upregulated compared to the vehicle-treated HRECs. Further analysis, using 1.5-fold change as a cutoff for significantly changed miRNAs, identified 29 downregulated and 45 upregulated miRNAs (Table 1).

### 3.2. Differential Expression Profile of miRNAs in HRECs Treated with High Glucose Compared to Control

**Figure 3:**
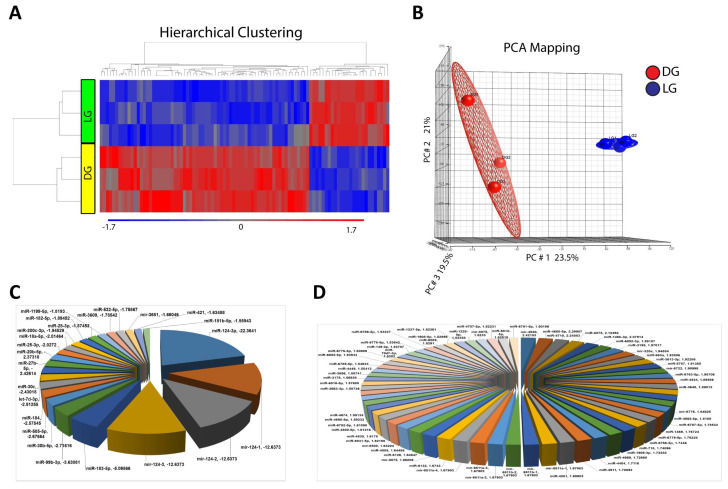
High Glucose Treatment Induces alteration of the miRNA profile of HRECs. Heat map demonstrating differential expression of miRNAs in osmotic control versus high glucose-treated HRECs. The blue color denotes downregulated miRNAs, and the red color signifies upregulated miRNAs (**A**). PCA mapping shows a distinction between different groups with less variation within the experiment (**B**). Pie chart demonstrating downregulated (**C**) and upregulated (**D**) miRNAs in HRECs in response to high glucose treatment.

To explore whether 15-HETE mimics the effects of hyperglycemia on retinal vasculature, HRECs were treated with high glucose (DG) or its osmotic and metabolic control (LG) for 5 days. High glucose treatment induced a distinct miRNA expression profile in HRECs compared to the control (Figure 3). Microarray analysis of RNA isolated from HRECs showed that 185 miRNAs were significantly downregulated, while 244 miRNAs were significantly upregulated under hyperglycemic conditions versus control. Using a 1.5-fold change cutoff, 24 miRNAs were found to be significantly downregulated, and 64 miRNAs were significantly upregulated (Table 2).

### 3.3. Comparison of miRNAs Commonly Changed in HRECs Challenged with 15-HETE or HG

**Figure 4:**
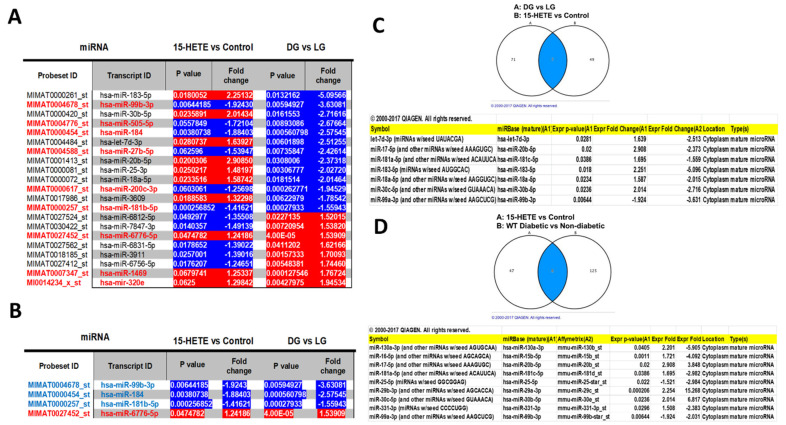
Comparison of miRNAs commonly changed in HRECs challenged with 15-HETE for 24 h or HG for 5 days. Multiple common miRNAs that significantly changed under both conditions (**A**). miRNAs consistently altered in HRECs following treatment with 15-HETE or DG (**B**). IPA identified seven miRNAs that are commonly altered in both DG and 15-HETE-treated HRECs (**C**). IPA identified nine miRNAs commonly altered by 15-HETE-treated HRECs and diabetic mouse retinas (**D**).

We hypothesized that 15-HETE induces miRNA alteration in endothelial cells—similar to the effects seen with high glucose. To test this hypothesis, we compared the datasets from both conditions and identified multiple common miRNAs that significantly changed under both conditions (Figure 4A). Through further analysis, we identified a core set of four miRNAs consistently altered in HRECs following treatment with 15-HETE or DG. Three miRNAs (miR-99, miR-184, miR-181) were downregulated under both conditions, while one miRNA (miR-6776) was upregulated under both conditions. This shortlist of four miRNAs represents promising targets for future-focused studies (Figure 4B). Furthermore, we used the IPA tool to identify the miRNAs that are commonly changed in HRECs challenged with 15-HETE for 24 h or HG for 5 days. IPA identified seven miRNAs (let-7d-3P, miR-17, miR-181, mi183, miR-18, miR-30, miR-99) that were commonly altered in both DG and 15-HETE-treated HRECs (Figure 4C).

### 3.4. Comparison of miRNAs Commonly Changed by 15-HETE-Treated HRECs and Diabetic Mouse Retinas

In this comparison, we aimed to identify similarities in the miRNA profiles of HRECs treated with 15-HETE and our previously published miRNA profile of diabetic mouse retina [12]. Using IPA, we identified nine miRNAs (miR-130, miR-16, miR-17, miR-181, miR-25, miR-29, miR-30, miR-331, miR-99) that were commonly altered under both conditions. These miRNAs are (Figure 4D). If we were to further compare 15-HETE-treated HRECs with HRECs treated with DG and diabetic mouse retinas, miR-181, miR-30, and miR-99 emerge as potential targets for future research.

### 3.5. Mirwalk2 Analysis for miRNAs of HRECs Treated with 15-HETE for 24 h

**Figure 5:**
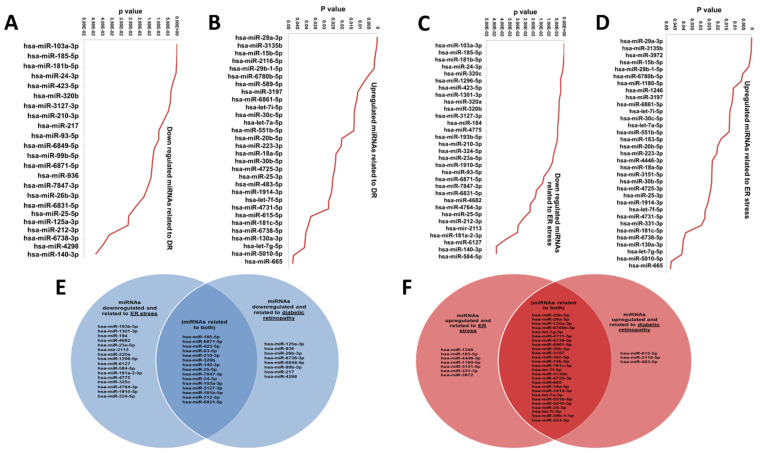
Mirwalk2 analysis of miRNAs in HRECs treated with 15-HETE for 24 h, demonstrating downregulated (**A**) and upregulated (**B**) miRNAs involved in DR as well as downregulated (**C**) and upregulated (**D**) miRNAs related to ER stress. Commonly downregulated (**E**) and upregulated (**F**) miRNAs are shown in Venn diagrams.

We used miRWalk2.0, a comprehensive atlas of microRNA–target interactions, as an initial analysis method for our dataset. We focused on miRNAs involved in DR (Figure 5A,B). We also used this tool to identify miRNAs involved in ER stress, which we previously linked to lipid-induced endothelial dysfunction during DR [10] (Figure 5B,C). This analysis highlighted possible target genes affected—the disturbed lipid metabolism induced by diabetes in HRECs. 15-HETE treatment downregulated miRNAs involved in DR, potentially affecting their suggested target genes (Table 3). Conversely, another group of miRNAs related to DR pathogenesis was found to be upregulated, potentially disturbing their target genes related to DR (Table 4). Given our interest in ER stress as a mechanism of 12/15-HETE-induced retinal endothelial dysfunction, we detected miRNAs associated with the ER stress and their possible affected target genes, both those that were downregulated (Table 5) and the upregulated (Table 6). We further showed the common miRNAs related to both DR and ER stress, including both upregulated and downregulated miRNAs (Figure 5E,F).

### 3.6. Differential Expression of HREC-Derived Exosomal-miRNAs After 15-HETE Treatment

**Figure 6:**
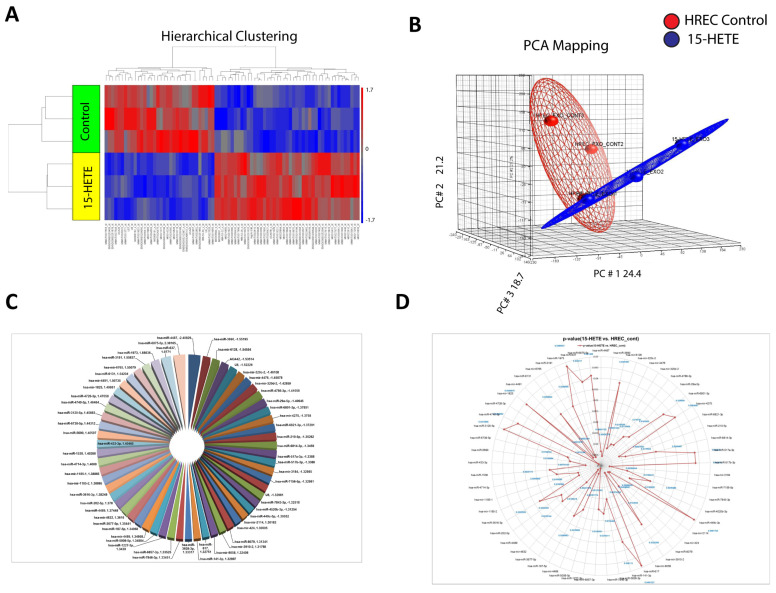
miRNA microarray for RNA isolated from EVs released from HRECs treated with 15-HETE (0.1 µM) for 24 h. Heat map demonstrating differential expression of EV-derived miRNAs in control versus 15-HETE-treated HRECs. The blue color denotes downregulated miRNAs, and the red color signifies upregulated miRNAs (**A**). PCA mapping showing distinction between different groups with less variation within the experiment (**B**). Pie chart demonstrating significantly changed miRNAs (**C**). *p*-values are diagrammed (**D**).

To explore if 15-HETE induces changes in the miRNA content of HREC-derived exosomes as a possible early intercellular communication tool that can deteriorate retinal barrier function, FBS-free conditioned media of HRECs cells pre-treated with 15-HETE (0.1 μM) for 24 h were collected. Exosomes were isolated using the described protocol in the methods section. The quality and quantity of exosomal miRNA have not been affected by the method of exosomal isolation [25,27]. The NTA of isolated exosomes showed the expected size range (40–150 nm) and identified the exosome concentration in each sample. RNA isolated from these exosomes underwent miRNA microarray analysis, revealing a significant impact of 15-HETE on the miRNA cargo of HREC-derived exosomes (Figure 6). Using a 1.3-fold change cutoff, we identified 79 miRNAs (34 were downregulated and 45 were upregulated) in exosomes derived from 15-HETE-treated HRECs compared to the control (Table 7).

### 3.7. IPA Analysis of Exosomal miRNAs Derived from HRECs Treated with 15-HETE for 24 h

We explored different pathways involved in 15-HETE-induced endothelial dysfunction, which could be linked to miRNA contents of endothelial-derived exosomes. IPA analysis revealed that exosomal miRNAs released from HRECs are involved in VEGF and angiogenesis signaling pathways. Moreover, they were associated with hypoxia and HIF1a signaling with phosphatase and tensin homolog (PTEN) appearing as a recurrent target for these exosomal miRNAs. The eNOS and iNOS signaling pathways were also identified as potential targets affected by changes in endothelial exosomal miRNA cargo due to 15-HETE treatment. Our IPA analysis also identified the involvement of other cellular pathways, such as ER stress, AMPK signaling, and inflammasome pathways, which were also detected. The mTOR signaling pathway, along with pathways related to autophagy and apoptosis, were also affected, indicating multiple possible targets for the released HREC exosomal miRNAs (Table 8).

## 4. Discussion

We have previously established the key role of 12/15-LO as a potential key contributor to diabetes-induced endothelial microvascular dysfunction in DR mediated through NADPH oxidase, VEGFR2 signaling, and ER stress [9,10]. The current study extends this understanding by underscoring an additional avenue through which 12/15-LO-derived metabolites regulate various signaling pathways implicated in the pathogenesis of endothelial cell dysfunction in DR. Specifically, we uncovered the potential involvements of miRNAs as significant players in retinal endothelial dysfunction induced by 12/15-LO-derived metabolites in DR. Through comprehensive microarray studies, we investigated differentially expressed cellular and exosomal miRNAs influenced by the 12/15-LO metabolite 15-HETE. Furthermore, we compared the miRNA profiles of endothelial cells following 15-HETE treatment to those under high glucose conditions and in the retina of diabetic mice. Our meticulous analysis of the altered miRNA expression patterns led us to identify a subset of miRNAs commonly dysregulated under both treatments, resulting in the formulation of a proposed shortlist of miRNAs. This shortlist could be a possible future area of research that may unveil novel diagnostic and therapeutic pathways in DR. 

Our major findings of this study are the following: (1) Exploring the commonly changed retinal endothelial miRNAs under high glucose or 15-HETE treatment revealed four miRNAs, three of which were downregulated, namely, miR-99b-5p, miR-184, and miR-181b-5p, and one of which was upregulated, namely, miR-6776-5p; (2) When comparing the results of 15-HETE-treated HRECs to diabetic mouse retinas, five miRNAs were commonly changing, of which, three were upregulated (miR-20b-5p, miR-29a-3p, and miR-30b-5p) and two were downregulated (miR-25-5p and miR-99b-3p; (3) The similarity between several miRNAs patterns confirms the importance of lipoxygenase product (15-HETE) in triggering DR phenotype in HRECs and highlights the most essential miRNAs of interest for future research.

In the current study, we found that hsa-miR-99b-3p was commonly downregulated in all comparisons. miR-99b was shown to target the mTOR, NF-κB, and AKT signaling pathways [28]. Interestingly, Hildebrand et al. [29] reported that miR-99b forms clusters with Let-7e and miR-125a, which in turn stabilize the suppressive function of antigen-presenting cells (APCs). This is partially achieved by supporting the STAT3-mediated expression of anti-inflammatory factors such as programmed death ligand (PDL)-1 and indolamine-2, 3-dioxygenase (IDO). The function of miR 99b as a crucial player in immunosuppression explains its possible role in the DR context since the DR is characterized by inflammation; therefore, a decrease in the anti-inflammatory key players such as miR 99b would be an exaggerating factor in the inflammatory response. 

Interestingly, the healing ability of exosomes derived from hypoxic adipose stem cells was attributed to a specific miRNA profile, which involved the downregulation of miR-99b, compared to normoxic conditions [30]. This downregulation contributes, in part, to the enhancement of the proliferation and migration of fibroblasts and the regulation of immune response [31]. Whether miR-99b-3p functions as an anti-inflammatory or its downregulation contributes to wound healing depends on cluster formation and the disease model, which requires further research.

Of note, the contribution of miR-99b to the pathogenesis of diabetic neuropathy and nephropathy has been reported [32,33]. miR-99b-5p, together with other miRNAs, was shown to mediate the neuroprotective effect of ischemic preconditioning against transient cerebral ischemia in a diabetic animal model [34]. The miR-99b family was repeatedly involved in different cancers, such as ovarian cancer, squamous cell carcinoma, and endometrial cancer, with debatable results about its role as either a tumor promoter or suppressor [35,36,37]. Implication in polycystic ovary syndrome, rheumatoid arthritis, and chronic fatigue syndrome was also reported [38,39,40].

Regarding hsa-miR-184, we noticed common downregulation in both DG and 15-HETE-treated HRECs by 1.9 and 2.6 folds, respectively. Consistent with our findings, Aykutlu et al. [41] highlighted its protective role in in-vitro models of age-related macular degeneration (AMD) through suppression of apoptosis, DNA damage, and angiogenesis, which alleviates hypoxia and oxidative-stress-mediated consequences. miR-184 was confirmed to enhance the differentiation of pluripotent stem cells to retinal pigment epithelial (RPE) cells through inhibition of the AKT/mTOR pathway [42]. Taken together, the downregulation of miR-184 in our models may contribute to the pathology of DR. The disrupted function of miR-184 in cellular proliferation may be contributing to the blood–retinal barrier dysfunction in DR.

Interestingly, tumor suppressor properties of miR-184 were confirmed in different studies. For instance, low expression of miR-184 was shown to promote tumor aggressiveness in malignant glioma cell lines and tissues [43]. In contrast, miR-184 was stated to be regulated by SNHG11, which belongs to long non-coding RNAs (lncRNAs); thus, its decrease resulted in decreased cellular proliferation, migration, and enhanced apoptosis in hepatocellular carcinoma (HCC) [44]. miR-184 was reported to be an important diagnostic and prognostic marker for non-small cell lung cancer (NSCLC) [45]. Another study revealed its involvement in competing endogenous RNA (ceRNA) networks in hypertrophic cardiomyopathy patients [46]. 

In the current study, hsa-miR-181b-5p was downregulated in both comparisons (DG vs. LD and 15-HETE-treated vs. CTRL HRECs). In agreement with this finding, Wang and Yu [47] emphasized the protective role of miR-181d-5p in high-glucose-treated HRECs by targeting VEGFA. Similarly, Yang et al. [48] demonstrated that miR-181a inhibits VEGF expression and hence, decreases neovascularization in different models.

In contrast to our findings, miR-181 displayed high levels in DR patients’ plasma and aqueous humor. Moreover, it boosted the proliferation and migration of retinal endothelial cells by targeting Kruppel-like factor (KLF)-6 [49]. Also, the downregulation of the miR-181 family was reported to ameliorate mitochondrial diseases of the retina, such as Leber’s hereditary optic neuropathy, through the balanced promotion of mitochondrial biogenesis and mitophagy [50]. Variations between the disease models and study settings in different studies may explain the contradictory results. 

Interestingly, to our knowledge, hsa-miR 6776-5P has not been investigated well so far. Of note, hsa-miR 6776-5P was upregulated in our study. Searching for predictive targets of miR-6776 revealed very interesting findings. A total of 5315 transcripts were predictive targets for this miRNA. ALOX15 (12/15-LO) and ALOX5 (5-LO) are predictive targets, which may represent a novel regulation mechanism of these important lipid-regulating enzymes in the retina. Moreover, miR-6776 can target antioxidant enzymes such as superoxide dismutase and glutathione peroxidase, which may contribute to the oxidative stress with reactive oxygen species generation observed in DR. Retinal barrier dysfunction in DR may be attributed to disrupted cell adhesion molecules such as cadherins and occludins [51].

Intriguingly, Occludin, Cadherin 4, and ICAM 1 are among the predictive targets of miR-6776. Hypoxia-related genes are also predicted to be targets for miR-6776, such as hypoxia-inducible factor 1, alpha (HIF-1α), and its inhibitor. Another interesting target for retina research is guanylate cyclase activator B in the retina. Retinal guanylate cyclase plays a vital function in photoreceptor cells during light response. Mutations in genes coding this protein were associated with certain types of blindness [52,53].

Comparing the differentially expressed miRNAs in HRECs treated with 15-HETE to miRNAs changed in diabetic mouse retinas revealed upregulation of hsa-miR-20b-5p in both data sets by 2.9 and 3.8 folds, respectively. Similar to our findings, Zhu et al. [54] confirmed that the proliferative fibro-vascular membranes from patients with DR exhibited high levels of miR-20b-5p. They also showed that hsa-miR-20b-5p triggers the proliferation, migration, and tube formation in HRECs under diabetic conditions. The high levels of miR-20b-5P were attributed to the downregulation of circDNMT3B, which functions as a sponge for miR-20b-5P.

In contrast to our findings, miR-20b-5p was downregulated in the sera of patients with DR as well as in ARPE-19 cells treated with HG. High levels of miR-20b-5p supported the proliferation and decreased the apoptosis and pyroptosis of ARPE-19 cells by targeting STAT3 [55]. A protective role of miR-20b-5p was also described by Wang et al. [56] in retinoblastoma cells by targeting STAT3, resulting in inhibited proliferation and enhanced apoptosis of tumor cells.

Upregulation of hsa-miR-29a-3p in both 15-HETE-treated HRECs and diabetic mouse retinas was observed. Consistent with this finding, miR-29a-3p upregulation was reported in the sera of patients with neovascular age-related macular degeneration (AMD) [57]. Overexpression of miR-29a-3p was shown to heighten STZ-induced retinal pericyte degeneration and vascular dysfunction [58]. Nonetheless, this miRNA was reported to be downregulated in human retinal microvascular endothelial cells under angiogenic stimulation and was linked to the regulation of apoptotic signaling [59]. Our recent study reported that miR-29a was able to attenuate the 12-HETE-induced inflammation and oxidative stress in retinal Muller cells [60]. Intriguingly, overexpression of miR-29a-3p was marked as an activator of protective autophagy by targeting Akt3/mTOR in transforming growth factor (TGF)-β-treated TC-1 cells, as a model of lung fibrosis, and resulted in amelioration of lung fibrosis and protection of lung epithelial cells [61]. Furthermore, overexpression of miR-29a-3p was also reported to inhibit malignant transformation in the occupational lung cancer model [62]. miR-29 was also reported to attenuate pathological retinal neovascularization and stroke-associated neuronal injury [60].

Moreover, hsa-miR-30b-5p was upregulated in both 15-HETE-treated HRECs and diabetic mouse retina. This upregulation was even more obvious in WT diabetic mice, where they exhibited a 6.8-fold increase compared to WT non-diabetic mice. Interestingly, Mazzeo and colleagues [63,64] reported upregulation of miR-30b-5p in EVs isolated from the sera of diabetic patients with DR compared to controls. Treatment of human retinal pericytes (HRP) with the aforementioned EVs, or transfection of HRP with miR-30b-5p mimics resulted in enhanced detachment and migration of pericytes, increased barrier dysfunction, and vessel-like structures formation, in comparison to EVs derived from control subjects. Further, a pro-angiogenic role of miR-30b-5p, shuttled by mesenchymal stem-cell-derived EVs, was also described [65].

Furthermore, miR-25-5p displayed downregulation in both 15-HETE-treated HRECs and diabetic mouse retinas by 1.5 and 3 folds, respectively. It was proposed to play a critical role in microvascular disorders. In line with our findings, treatment of human brain microvessel endothelial cells (HBMECs) with oxidized low-density lipoproteins (ox-LDL) caused decreased expression of miR-25-5p, which, when overexpressed, neutralized the effect of ox-LDL, with decreased apoptosis, reactive oxygen species (ROS), and nitric oxide (NO) production [66]. Additionally, miR 25-5p activation has been described in the mechanism of action of some anticancer treatment regimens in colorectal cancer and oral squamous cell carcinoma models [67,68,69].

After investigating changes in endothelial cellular miRNAs under different conditions, we turned our attention to examining the changes in endothelial-derived exosomal miRNAs. Exosomal miRNAs play a crucial role in intercellular communication within the retina during DR. As retinal endothelial cells are the initial site within the retina exposed to elevated glucose levels during diabetes, we hypothesized that these cells might transmit signals to neighboring retinal cells via released exosomes in response to hyperglycemic stress. Exosomal miRNAs are proposed as a mechanism for conveying these signals from the affected endothelium to other retinal cells. 

## 5. Conclusions

Our microarray studies demonstrated multiple novel miRNAs that could represent new diagnostic and therapeutic targets in DR. Changes in the miRNA profile of the retinal endothelial cells after 15-HETE treatment showed some similarities to miRNA profile changes induced by hyperglycemia. These similarities highlight the pivotal role of the 12/15 lipoxygenase pathway and its inflammatory lipid mediators in the pathogenesis of DR.

## Figures and Tables

**Table 1:** Differential expression profile of miRNAs in HRECs treated with 15-HETE.

	Transcript ID (Array Design)	Fold-Change (15-HETE vs. Control) Downregulated miRNAs
1	miR-193b-5p	−2.13941
2	miR-185-5p	−2.12926
3	miR-99b-3p	−1.9243
4	miR-1301-3p	−1.92395
5	miR-184	−1.88403
6	miR-6871-5p	−1.87815
7	miR-4682	−1.78805
8	miR-125a-3p	−1.77299
9	miR-574-3p	−1.66244
10	miR-423-5p	−1.65963
11	miR-936	−1.64587
12	miR-93-5p	−1.61362
13	miR-23a-5p	−1.57997
14	mir-6770-1	−1.57223
15	mir-6770-2	−1.57223
16	mir-6770-3	−1.57223
17	miR-210-3p	−1.57068
18	miR-7854-3p	−1.568
19	miR-659-3p	−1.56316
20	miR-320b	−1.56315
21	miR-140-3p	−1.5526
22	mir-2113	−1.55132
23	miR-320a	−1.54671
24	miR-651-5p	−1.54507
25	miR-1296-5p	−1.52955
26	miR-26b-3p	−1.52905
27	miR-25-5p	−1.52137
28	miR-6738-3p	−1.51287
29	miR-4730	−1.50367
	**Transcript ID (Array Design)**	**Fold-Change (15-HETE vs. Control) Upregulated miRNAs**
1	miR-20b-5p	2.9085
2	miR-1246	2.90223
3	miR-29a-3p	2.25368
4	miR-183-5p	2.25132
5	miR-130a-3p	2.20103
6	miR-6780b-5p	2.18853
7	U71d	2.18837
8	let-7g-5p	2.1858
9	miR-4731-5p	2.15909
10	miR-6738-5p	2.12149
11	miR-6861-5p	2.01633
12	miR-30b-5p	2.01434
13	miR-6815-5p	1.99778
14	miR-615-5p	1.95037
15	miR-3197	1.93633
16	miR-4485	1.82265
17	miR-30c-5p	1.81731
18	miR-4446-3p	1.80924
19	U49B	1.80587
20	miR-1180-5p	1.73416
21	miR-15b-5p	1.72064
22	miR-181c-5p	1.69496
23	let-7f-5p	1.69172
24	U38B	1.66528
25	miR-2116-5p	1.6609
26	U42A	1.65613
27	let-7d-3p	1.63927
28	U46	1.63072
29	miR-1238-5p	1.60282
30	miR-3135b	1.59757
31	miR-3151-5p	1.59704
32	miR-4725-3p	1.5942
33	miR-483-5p	1.59252
34	miR-665	1.59135
35	miR-18a-5p	1.58742
36	miR-1914-3p	1.58741
37	U27	1.58432
38	U78	1.57467
39	let-7a-5p	1.5496
40	miR-6877-5p	1.53461
41	miR-3909	1.51443
42	miR-331-3p	1.5079
43	U18A	1.50782
44	miR-551b-5p	1.50401
45	miR-5010-5p	1.50013

**Table 2:** Differential expression profile of miRNAs in HRECs treated with high glucose compared to control.

	Transcript ID (Array Design)	Fold-Change (DG vs. LG) Downregulated miRNAs
1	miR-124-3p	−22.3641
2	mir-124-1	−12.6373
3	mir-124-2	−12.6373
4	mir-124-3	−12.6373
5	miR-183-5p	−5.09566
6	miR-99b-3p	−3.63081
7	miR-30b-5p	−2.71616
8	miR-505-5p	−2.67664
9	miR-184	−2.57545
10	let-7d-3p	−2.51255
11	miR-30c	−2.43018
12	miR-27b-5p	−2.42614
13	miR-20b-5p	−2.37318
14	miR-25-3p	−2.0272
15	miR-18a-5p	−2.01464
16	miR-200c-3p	−1.94529
17	miR-182-5p	−1.89452
18	miR-28-3p	−1.87453
19	miR-1199-5p	−1.8193
20	miR-3609	−1.78542
21	miR-532-5p	−1.75867
22	mir-3651	−1.66046
23	miR-421	−1.63488
24	miR-181b-5p	−1.55943
	**Transcript ID (Array Design)**	**Fold-Change (DG vs. LG) Upregulated miRNAs**
1	mir-4530	2.42153
2	miR-4668-5p	2.29957
3	miR-4710	2.24883
4	miR-8075	2.12494
5	miR-146b-3p	2.07914
6	miR-6892-3p	1.99157
7	miR-3195	1.97017
8	mir-320e	1.94534
9	miR-663a	1.93806
10	miR-3613-3p	1.92286
11	miR-5787	1.91385
12	mir-6722	1.90995
13	miR-6763-5p	1.90706
14	miR-4534	1.89586
15	miR-3648	1.89012
16	mir-6776	1.84825
17	miR-4665-5p	1.8185
18	miR-6787-5p	1.78533
19	miR-1469	1.76724
20	miR-6779-5p	1.76228
21	miR-6756-5p	1.7446
22	miR-718	1.74096
23	miR-1909-3p	1.73232
24	miR-4669	1.72668
25	miR-4484	1.7116
26	miR-3911	1.70093
27	miR-4281	1.69903
28	mir-6511a-1	1.67903
29	mir-6511b-1	1.67903
30	mir-6511b-2	1.67903
31	mir-6511a-2	1.67903
32	mir-6511a-3	1.67903
33	mir-6511a-4	1.67903
34	miR-6132	1.6743
35	mir-8075	1.66889
36	miR-6126	1.64547
37	miR-4505	1.64455
38	mir-6500	1.63224
39	miR-6831-5p	1.62166
40	miR-4530	1.6175
41	miR-4800-5p	1.61216
42	miR-6782-5p	1.61098
43	miR-4690-5p	1.59233
44	miR-4674	1.59134
45	miR-3663-3p	1.58736
46	miR-6816-5p	1.57688
47	miR-3178	1.56938
48	miR-3656	1.55741
49	miR-4449	1.55412
50	miR-6789-5p	1.54633
51	miR-6803-5p	1.53944
52	miR-6776-5p	1.53909
53	miR-7847-3p	1.5382
54	miR-149-3p	1.53707
55	miR-6778-5p	1.53642
56	miR-6786-5p	1.53337
57	miR-6085	1.5291
58	miR-1908-5p	1.52896
59	miR-1237-5p	1.52361
60	miR-1228-5p	1.52358
61	mir-8075	1.5233
62	miR-4707-5p	1.52231
63	miR-6812-5p	1.52015
64	miR-6791-5p	1.50186

**Table 3:** Mirwalk2 analysis for miRNAs of HRECs treated with 15-HETE. Downregulated miRNAs with target genes involved in DR.

miRNA (Downregulated in Our Model)	*p*-Value	Fold Change	Target Genes Involved in Diabetic Retinopathy (miRWalk2.0: a Comprehensive Atlas of microRNA–Target Interactions)
hsa-miR-185-5p	7.83497 × 10^−5^	−2.12926	KITLG, SOD2, VEGFA
hsa-miR-6871-5p	0.0130272	−1.87815	SP1, SOD2
hsa-miR-125a-3p	0.0259088	−1.77299	MTHFR, LIPG
hsa-miR-423-5p	0.00224777	−1.65963	SP1, TIMP3
hsa-miR-936	0.0135351	−1.64587	FGF2, SOD2
hsa-miR-93-5p	0.00969692	−1.61362	SOD2, HIF1A, ICAM1, ITGA2, PRKCB, VEGFA, SOD2
hsa-miR-210-3p	0.00459651	−1.57068	HIF1A
hsa-miR-320b	0.00335084	−1.56315	MAPK3
hsa-miR-140-3p	0.0436274	−1.5526	GDNF
hsa-miR-26b-3p	0.0157183	−1.52905	FGF2
hsa-miR-25-5p	0.0219829	−1.52137	ENG
hsa-miR-6738-3p	0.0364529	−1.51287	VASH1, EDN1
hsa-miR-7847-3p	0.0140357	−1.49139	IGF1, SOD2, MTHFR
hsa-miR-6849-5p	0.00988734	−1.46607	SOD2
hsa-miR-99b-5p	0.0123929	−1.46407	SP1, ENO2
hsa-miR-24-3p	0.000539484	−1.46219	IGF1, LIPG, NOS3, CCL2
hsa-miR-103a-3p	3.36016 × 10^−6^	−1.44571	ITGA2, MTHFR, TIMP3, FGF2
hsa-miR-3127-3p	0.00351928	−1.42848	SP1, APLN
hsa-miR-217	0.00715815	−1.41702	HIF1A
hsa-miR-181b-5p	0.000256852	−1.41621	TIMP3, PRKCD, VCAM1
hsa-miR-212-3p	0.0262645	−1.40178	SOD2
hsa-miR-4298	0.0396148	−1.39616	SOD2
hsa-miR-6831-5p	0.0178652	−1.39022	EDN1, FGF2

**Table 4:** Mirwalk2 analysis for miRNAs of HRECs treated with 15-HETE. Upregulated miRNAs with target genes involved in DR.

miRNA (Downregulated in Our Model)	*p*-Value	Fold Change	Target Genes Involved in ER Stress (miRWalk2.0: a Comprehensive Atlas of microRNA–Target Interactions)
hsa-miR-193b-5p	0.0042701	−2.13941	DNAJC10, CHAC1
hsa-miR-185-5p	7.83497 × 10^−5^	−2.12926	GFPT1, DNAJC10, CREB3L2
hsa-miR-1301-3p	0.00226551	−1.92395	VCP, HDGF, EDEM1
hsa-miR-184	0.00380738	−1.88403	BCL2, VIMP
hsa-miR-6871-5p	0.0130272	−1.87815	CHAC1, ATF6
hsa-miR-4682	0.0185978	−1.78805	TATDN2
hsa-miR-423-5p	0.00224777	−1.65963	BAK1
hsa-miR-93-5p	0.00969692	−1.61362	SCAMP5, FAM129A, DNAJC10, CREBRF, XBP1
hsa-miR-23a-5p	0.00695373	−1.57997	SSR1, HDGF
hsa-miR-210-3p	0.00459651	−1.57068	PTPN1
hsa-miR-320b	0.00335084	−1.56315	CREBRF, YOD1
hsa-miR-140-3p	0.0436274	−1.5526	GFPT1, AMFR, PPP1R15A
hsa-mir-2113	0.0296329	−1.55132	GFPT1
hsa-miR-320a	0.00314374	−1.54671	CREBRF, XBP1, YOD1, TSPYL2, DNAJB9, CALR
hsa-miR-1296-5p	0.00222623	−1.52955	DCTN1, HYOU1, VCP
hsa-miR-25-5p	0.0219829	−1.52137	HSP90B1
hsa-miR-7847-3p	0.0140357	−1.49139	CALR, HDGF, CHAC1, DNAJC10, COL4A3BP
hsa-miR-6127	0.0375442	−1.47443	CTDSP2, HYOU1, CREB3L2
hsa-miR-24-3p	0.000539484	−1.46219	ADD1, ATF3, CCL2, CCND1, CTDSP2, DNAJC3, ERO1L, IFNG, KLHDC3, SSR1, TLN1, YOD1, DNAJC10
hsa-miR-584-5p	0.0436807	−1.46208	UBE4B, HSPA5
hsa-miR-181a-2-3p	0.0299875	−1.45872	YOD1, KLHDC3, DNAJC3
hsa-miR-103a-3p	3.36016 × 10^−6^	−1.44571	ERN1, BCL2, DNAJC10, CREBRF
hsa-miR-4775	0.00417953	−1.43377	DNAJC10
hsa-miR-3127-3p	0.00351928	−1.42848	HSPA5
hsa-miR-320c	0.00102898	−1.41974	CREBRF, YOD1
hsa-miR-181b-5p	0.000256852	−1.41621	BCL2, DNAJB11, FKBP14, HSP90B1, PDIA6
hsa-miR-4764-3p	0.0218295	−1.41492	DNAJC3
hsa-miR-1910-5p	0.00757886	−1.41238	CALR
hsa-miR-212-3p	0.0262645	−1.40178	CHAC1
hsa-miR-324-5p	0.00586432	−1.39873	DDX11, KLHDC3, YOD1
hsa-miR-6831-5p	0.0178652	−1.39022	YOD1, HERPUD1

**Table 5:** Mirwalk2 analysis for miRNAs of HRECs treated with 15-HETE. Downregulated miRNAs with target genes involved in ER stress.

miRNA (Downregulated in Our Model)	*p*-Value	Fold Change	Target Genes Involved in ER Stress (miRWalk2.0: a Comprehensive Atlas of microRNA–Target Interactions)
hsa-miR-193b-5p	0.0042701	−2.13941	DNAJC10, CHAC1
hsa-miR-185-5p	7.83497 × 10^−5^	−2.12926	GFPT1, DNAJC10, CREB3L2
hsa-miR-1301-3p	0.00226551	−1.92395	VCP, HDGF, EDEM1
hsa-miR-184	0.00380738	−1.88403	BCL2, VIMP
hsa-miR-6871-5p	0.0130272	−1.87815	CHAC1, ATF6
hsa-miR-4682	0.0185978	−1.78805	TATDN2
hsa-miR-423-5p	0.00224777	−1.65963	BAK1
hsa-miR-93-5p	0.00969692	−1.61362	SCAMP5, FAM129A, DNAJC10, CREBRF, XBP1
hsa-miR-23a-5p	0.00695373	−1.57997	SSR1, HDGF
hsa-miR-210-3p	0.00459651	−1.57068	PTPN1
hsa-miR-320b	0.00335084	−1.56315	CREBRF, YOD1
hsa-miR-140-3p	0.0436274	−1.5526	GFPT1, AMFR, PPP1R15A
hsa-mir-2113	0.0296329	−1.55132	GFPT1
hsa-miR-320a	0.00314374	−1.54671	CREBRF, XBP1, YOD1, TSPYL2, DNAJB9, CALR
hsa-miR-1296-5p	0.00222623	−1.52955	DCTN1, HYOU1, VCP
hsa-miR-25-5p	0.0219829	−1.52137	HSP90B1
hsa-miR-7847-3p	0.0140357	−1.49139	CALR, HDGF, CHAC1, DNAJC10, COL4A3BP
hsa-miR-6127	0.0375442	−1.47443	CTDSP2, HYOU1, CREB3L2
hsa-miR-24-3p	0.000539484	−1.46219	ADD1, ATF3, CCL2, CCND1, CTDSP2, DNAJC3, ERO1L, IFNG, KLHDC3, SSR1, TLN1, YOD1, DNAJC10
hsa-miR-584-5p	0.0436807	−1.46208	UBE4B, HSPA5
hsa-miR-181a-2-3p	0.0299875	−1.45872	YOD1, KLHDC3, DNAJC3
hsa-miR-103a-3p	3.36016 × 10^−6^	−1.44571	ERN1, BCL2, DNAJC10, CREBRF
hsa-miR-4775	0.00417953	−1.43377	DNAJC10
hsa-miR-3127-3p	0.00351928	−1.42848	HSPA5
hsa-miR-320c	0.00102898	−1.41974	CREBRF, YOD1
hsa-miR-181b-5p	0.000256852	−1.41621	BCL2, DNAJB11, FKBP14, HSP90B1, PDIA6
hsa-miR-4764-3p	0.0218295	−1.41492	DNAJC3
hsa-miR-1910-5p	0.00757886	−1.41238	CALR
hsa-miR-212-3p	0.0262645	−1.40178	CHAC1
hsa-miR-324-5p	0.00586432	−1.39873	DDX11, KLHDC3, YOD1
hsa-miR-6831-5p	0.0178652	−1.39022	YOD1, HERPUD1

**Table 6:** Mirwalk2 analysis for miRNAs of HRECs treated with 15-HETE. Upregulated miRNAs with target genes involved in ER stress.

miRNA (Upregulated in Our Model)	*p*-Value	Fold Change	Target Genes Involved in ER Stress (miRWalk2.0: a Comprehensive Atlas of microRNA–Target Interactions)
hsa-miR-20b-5p	0.0200306	2.9085	SCAMP5, FAM129A, YOD1, HDGF.EIF2S1
hsa-miR-1246	0.0108907	2.90223	CREBRF
hsa-miR-29a-3p	0.000205702	2.25368	BCL2, AMFR, CCND1, HDGF, KLHDC3, SEC31A, BBC3, BCAP31
hsa-miR-183-5p	0.0180052	2.25132	USP19, PREB, HYOU1, CCND1, ASNS, PSEN1
hsa-miR-130a-3p	0.0405385	2.20103	ATP6V0D1, TPP1
hsa-miR-6780b-5p	0.00543559	2.18853	DDX11, KLHDC3, TPP1, BBC3
hsa-let-7g-5p	0.0409584	2.1858	CCND1, HERPUD1, YOD1
hsa-miR-4731-5p	0.0274671	2.15909	CCND1, YOD1, DNAJC10
hsa-miR-6738-5p	0.0400795	2.12149	CALR, BAK1
hsa-miR-6861-5p	0.0116901	2.01633	HDGF
hsa-miR-30b-5p	0.0235891	2.01434	BCL2, YOD1, SRPR, SHC1
hsa-miR-3197	0.0110853	1.93633	SRPR
hsa-miR-30c-5p	0.01312	1.81731	AIFM1, SRPR, ARFGAP1
hsa-miR-4446-3p	0.0231141	1.80924	CHAC1
hsa-miR-1180-5p	0.00631992	1.73416	GFPT1, HSPA5
hsa-miR-15b-5p	0.00109533	1.72064	SCAMP5, DNAJC10, CREBRF, CHAC1, BCL2, SRPRB, SRPR, PDIA6, IFNG, HYOU1
hsa-miR-181c-5p	0.0386374	1.69496	BCL2, FKBP14, HSP90B1, PDIA6, BCL2
hsa-let-7f-5p	0.0273735	1.69172	CCND1, HERPUD1, YOD1
hsa-miR-3135b	0.000338555	1.59757	DNAJC10
hsa-miR-3151-5p	0.0234764	1.59704	CHAC1
hsa-miR-4725-3p	0.0240861	1.5942	BBC3, TPP1, KLHDC3, DDX11
hsa-miR-665	0.0476011	1.59135	BBC3, ERN1, KLHDC3, HSP90B1, DNAJB9, CTDSP2, CALR
hsa-miR-18a-5p	0.0233516	1.58742	BCL2, VCP, CCND1
hsa-miR-1914-3p	0.0258546	1.58741	BAK1, CALR
hsa-let-7a-5p	0.0131744	1.5496	ERN1, BCL2, YOD1, SYVN1, PREB, LMNA, CCND1
hsa-miR-331-3p	0.0295934	1.5079	BAG6, VAPB, SEC31A, ATF3
hsa-miR-551b-5p	0.0133219	1.50401	YOD1, VAPB
hsa-miR-5010-5p	0.0471213	1.50013	CALR
hsa-miR-3972	0.000521049	1.49324	CHAC1
hsa-miR-25-3p	0.0250217	1.48197	EDEM1, DNAJB9, DCTN1, ITPR1, SRPR, TLN1, BAK1, FAM129A
hsa-let-7i-5p	0.0127131	1.48001	YOD1, HERPUD1, CCND1
hsa-miR-29b-1-5p	0.0019697	1.40081	DNAJB9
hsa-miR-223-3p	0.0205097	1.39724	HSP90B1

**Table 7:** Differential expression of HREC-derived exosomal-miRNAs after 15-HETE treatment.

	Transcript ID (Array Design)	*p*-Value	Fold-Change (Exososmes_15-HETE vs. Control) Downregulated miRNAs
1	hsa-miR-4487	0.00661752	−2.40829
2	hsa-miR-3690	0.00438276	−1.55195
3	hsa-mir-6128	0.0387961	−1.54554
4	ACA42	0.0490747	−1.53514
5	U8	0.00166388	−1.52226
6	hsa-mir-320c-2	0.00831453	−1.46108
7	hsa-mir-4476	0.0108395	−1.45078
8	hsa-mir-320d-2	0.0110809	−1.42859
9	hsa-miR-4786-3p	0.016741	−1.41058
10	hsa-miR-29a-5p	0.0183503	−1.40645
11	HBII-52-30	0.0125513	−1.38304
12	ENSG00000239188	0.0172407	−1.38152
13	ACA26	0.000695604	−1.37988
14	hsa-miR-6801-3p	0.036956	−1.37851
15	hsa-mir-4275	0.0404561	−1.3758
16	hsa-miR-6821-3p	0.00927923	−1.37291
17	ENSG00000239095	0.0144574	−1.36681
18	hsa-miR-210-5p	0.0119924	−1.35262
19	hsa-miR-6814-3p	0.0259467	−1.3459
20	hsa-miR-517a-3p	0.043272	−1.3388
21	hsa-miR-517b-3p	0.043272	−1.3388
22	ENSG00000251860	0.035814	−1.33472
23	hsa-mir-3184	0.00395034	−1.32985
24	hsa-miR-7156-5p	0.0122472	−1.32981
25	U8	0.0479245	−1.32981
26	ENSG00000238798	0.00710618	−1.32814
27	hsa-miR-7843-3p	0.0236484	−1.32518
28	hsa-miR-4520b-3p	0.0143208	−1.31254
29	ENSG00000212347	0.0331977	−1.3101
30	ENSG00000268513	0.0331977	−1.3101
31	ENSG00000251878	0.0300967	−1.30726
32	ENSG00000252409	0.0269747	−1.30663
33	U57	0.0104552	−1.30431
34	hsa-miR-449c-3p	0.0491754	−1.30052
	**Transcript ID** **(Array Design)**	***p*-Value**	**Fold-Change (Exososmes_15-HETE vs. Control) Upregulated miRNAs**
1	hsa-mir-2114	0.00499041	1.30162
2	mgU6-53B	0.0386137	1.3021
3	HBII-52-22	0.0361741	1.30235
4	hsa-mir-424	0.00438885	1.30605
5	HBII-52-26	0.0425845	1.3122
6	hsa-miR-8079	0.0123932	1.31341
7	hsa-mir-3910-2	0.0325295	1.31768
8	ENSG00000201025	0.0165037	1.32395
9	hsa-mir-8058	0.00374253	1.32408
10	ENSG00000238544	0.00376291	1.32674
11	hsa-miR-617	0.0491327	1.32751
12	hsa-miR-141-3p	0.036113	1.32897
13	hsa-miR-3928-3p	0.0155404	1.33317
14	ACA67B	0.0119016	1.33334
15	hsa-miR-7846-3p	0.0219211	1.33451
16	hsa-miR-6857-3p	0.0199351	1.33525
17	ENSG00000252096	0.00802232	1.33957
18	hsa-miR-1227-3p	0.00133352	1.3439
19	hsa-miR-5008-5p	0.00391714	1.34884
20	hsa-mir-4489	0.0216809	1.34958
21	hsa-miR-187-5p	0.0260663	1.34968
22	hsa-miR-3677-5p	0.00231678	1.35441
23	hsa-mir-4632	0.0100516	1.3616
24	hsa-miR-4489	0.0239144	1.37448
25	hsa-miR-202-5p	0.0224492	1.378
26	ENSG00000202268	0.0466552	1.38082
27	hsa-miR-3616-3p	0.0322928	1.38249
28	hsa-mir-1185-2	0.0109365	1.38865
29	hsa-mir-1185-1	0.0109365	1.38865
30	hsa-miR-4714-3p	0.0205829	1.4008
31	hsa-miR-1538	0.0235173	1.40268
32	hsa-miR-433-3p	0.00703121	1.40465
33	hsa-miR-5690	0.0142904	1.40597
34	hsa-miR-6738-5p	0.0156754	1.44312
35	hsa-miR-3120-5p	0.0473806	1.45883
36	hsa-miR-4740-3p	0.0426094	1.46464
37	hsa-miR-4726-3p	0.0012146	1.47058
38	hsa-mir-1825	0.0440976	1.49861
39	hsa-mir-4491	0.00512187	1.50735
40	hsa-miR-6131	0.0299504	1.54204
41	hsa-mir-6765	0.00453891	1.55079
42	hsa-miR-3181	0.0300586	1.55837
43	hsa-miR-1973	0.0480557	1.68636
44	hsa-miR-637	0.039217	1.9771
45	hsa-miR-6875-5p	0.041488	2.38165

**Table 8:** IPA analysis of exosomal miRNAs derived from HRECs treated with 15-HETE for 24 h.

miRNA	IPA Analysis Target Genes	Fold Change
**Exosomal miRNAs involved in Hypoxia signaling**		
hsa-let-7b-5p	CSNK1D	−1.712
hsa-miR-5189-5p	TP53	1.782
hsa-miR-140-5p	VEGFA	1.001
hsa-miR-143-3p	MDM2	1.17
hsa-miR-5195-3p	MDM2	1.505
hsa-miR-155-5p	UBE2J1	1.722
hsa-miR-16-5p	HSP90B1, JUN, UBE2S, VEGFA	2.44
hsa-miR-20a-5p	CREB1, PTEN, VEGFA	2.295
hsa-miR-185-5p	AKT1	1.596
hsa-miR-3619-5p	ATF4, PTEN	−1.478
hsa-miR-222-3p	PTEN	1.363
hsa-miR-23a-3p	PTEN	−1.74
hsa-miR-31-5p	HIF1A	1.494
hsa-miR-494-3p	PTEN	1.464
**Exosomal miRNAs involved in HIF1a signaling**		
hsa-let-7b-5p	HRAS, KRAS, NRAS, Ras	−1.712
hsa-miR-99b-5p	FGFR3	1.364
hsa-miR-124-3p	MAPK14, PGF	1.404
hsa-miR-125b-5p	TP53	1.137
hsa-miR-5189-5p	AKT2, TP53	1.782
hsa-miR-5195-3p	IRS1, MAPK7, MDM2, MMP1	1.505
hsa-miR-16-5p	FGFR1, GRB2, JUN, MAPK3, VEGFA	2.44
hsa-miR-20a-5p	MMP3, VEGFA	2.295
hsa-miR-185-5p	AKT1	1.596
hsa-miR-31-5p	HIF1A	1.494
**Exosomal miRNAs involved in VEGF signaling**		
hsa-let-7b-5p	BCL2L1, HRAS, KRAS, NRAS, Ras	−1.712
hsa-miR-99b-5p	FGFR3	1.364
hsa-miR-124-3p	PGF, ROCK1	1.404
hsa-miR-5189-5p	AKT2	1.782
hsa-miR-138-5p	ROCK2	1.561
hsa-miR-5195-3p	IRS1	1.505
hsa-miR-155-5p	FOXO3	1.722
hsa-miR-16-5p	BCL2, FGFR1, GRB2, MAP2K1, MAPK3, RAF1, VEGFA	2.44
hsa-miR-20a-5p	BCL2, VEGFA	2.295
hsa-miR-181c-5p	BCL2, KRAS	−1.312
**Exosomal miRNAs involved in inhibition of angiogenesis**		
hsa-let-7b-5p	CASP3, TGFBR1, THBS1	−1.712
hsa-miR-124-3p	MAPK14	1.404
hsa-miR-125b-5p	TP53	1.137
hsa-miR-5189-5p	AKT2, TP53	1.782
hsa-miR-141-3p	MAP2K4	1.329
hsa-miR-155-5p	CD47	1.722
hsa-miR-16-5p	JUN, MAP2K4, VEGFA	2.44
hsa-miR-20a-5p	TGFBR2, VEGFA	2.295
hsa-miR-185-5p	AKT1	1.596
hsa-miR-92a-3p	MAP2K4	−1.498
**Exosomal miRNAs involved in eNOS signaling**		
hsa-let-7b-5p	CASP3	−1.712
hsa-miR-99b-5p	FGFR3	1.364
hsa-miR-124-3p	CAV1, DNM2, PGF, PRKD1	1.404
hsa-miR-5189-5p	AKT2	1.782
hsa-miR-5195-3p	CCNA2, IRS1	1.505
hsa-miR-155-5p	PRKCI	1.722
hsa-miR-16-5p	FGFR1, GRB2, HSP90B1, HSPA1A/HSPA1B, SLC7A1, VEGFA	2.44
hsa-miR-20a-5p	ESR1, VEGFA	2.295
hsa-miR-181c-5p	ESR1	−1.312
hsa-miR-182-5p	ADCY6	1.31
hsa-miR-185-5p	AKT1	1.596
hsa-miR-22-3p	ESR1	1.403
hsa-miR-222-3p	ESR1, PIK3R1	1.363
**Exosomal miRNAs involved in iNOS signaling**		
hsa-let-7b-5p	HMGA1, TLR4	−1.712
hsa-miR-124-3p	MAPK14, RELA	1.404
hsa-miR-155-5p	IKBKE, MYD88	1.722
hsa-miR-16-5p	HMGA1, JUN	2.44
hsa-miR-20a-5p	JAK1	2.295
hsa-miR-222-3p	FOS	1.363
**Exosomal miRNAs involved in ER stress**		
hsa-let-7b-5p	CASP3	−1.712
hsa-miR-125b-5p	CASP7	1.137
hsa-miR-127-3p	XBP1	−1.092
hsa-miR-133a-3p	CASP9	1.015
hsa-miR-16-5p	ATF6	2.44
hsa-miR-16-5p	HSP90B1	2.44
hsa-miR-3619-5p	ATF4	−1.478
hsa-miR-503-5p	ATF6	−1.274
**Exosomal miRNAs involved in AMPK signaling**		
hsa-let-7b-5p	CCND1, GYS1	−1.712
hsa-miR-99b-5p	FGFR3, MTOR, RPTOR	1.364
hsa-miR-124-3p	AK2, MAPK14	1.404
hsa-miR-5189-5p	AKT2	1.782
hsa-miR-5195-3p	CCNA2, IRS1	1.505
hsa-miR-155-5p	ARID2, CCND1, FOXO3	1.722
hsa-miR-16-5p	CCND1, FGFR1, GRB2, PPP2R5C	2.44
hsa-miR-20a-5p	CCND1, CDKN1A, CREB1	2.295
hsa-miR-185-5p	AKT1	1.596
hsa-miR-193b-3p	CCND1	1.77
hsa-miR-3619-5p	ATF4	−1.478
hsa-miR-222-3p	FOXO3, PIK3R1, PPP2R2A	1.363
hsa-miR-31-5p	PPP2R2A	1.494
hsa-miR-92a-3p	CDKN1A	−1.498
**Exosomal miRNAs involved in Inflammasome pathway**		
hsa-let-7b-5p	TLR4	−1.712
hsa-miR-155-5p	MYD88	1.722
hsa-miR-16-5p	PANX1	2.44
hsa-miR-20a-5p	CXCL8	2.295
**Exosomal miRNAs involved in Apoptosis**		
hsa-let-7b-5p	BCL2L1, CASP3, CCND1, CDK6, HRAS, KRAS, MYC, NRAS, Ras, SLC25A13, TGFBR1, TLR4, VIM	−1.712
hsa-miR-99b-5p	FGFR3, IGF1R, MTOR	1.364
hsa-miR-103a-3p	CCNE1, CDK6, CRKL, NFIA	1.681
hsa-miR-124-3p	AHR, AHRR, ALDH9A1, CDK2, CDK4, CDK6, CEBPA, CHP1, CYP1B1, DFFB, ELF4, ELK3, F11R, ITGB1, MAPK14, MYH9, NFATC1, NFIC, PARP16, PRKD1, RARG, RELA, ROCK1, SP1, STAT3, TJP2, TNFRSF21, TRIP11, TUBB6, VAMP3	1.404
hsa-miR-5189-5p	AKT2, TP53	1.782
hsa-miR-138-5p	ALDH1A2, TERT	1.561
hsa-miR-141-3p	CTNNB1, CYP1B1, MAP2K4, STAT5B, TGFB2, YAP1	1.329
hsa-miR-5195-3p	CCNA2, CDK4, DDR1, DFFA, F11R, IGF1R, IRS1, MAPK7, MDM2, MYC, PARP8, PPP3CA	1.505
hsa-miR-152-3p	CCKBR	−1.353
hsa-miR-155-5p	CCND1, CEBPB, CLDN1, CTNNB1, ETS1, FADD, FOXO3, GNA13, IKBKE, INPP5D, MYD88, PRKCI, RHOA, RIPK1, SOCS1, TAB2, TNFRSF10A, VAMP3	1.722
hsa-miR-16-5p	BCL2, CCND1, CCND3, CCNE1, CDK6, CHEK1, CLDN12, EGFR, FGFR1, GRB2, GSTM4, HSP90B1, IGF1, IGF1R, IGF2R, ITGA2, JUN, MAP2K1, MAP2K4, MAPK3, MCL1, NAPG, NFIA, PDCD6IP, PPP2R5C, RAF1, VTI1B	2.44
hsa-miR-20a-5p	BCL2, BCL2L11, BMPR2, CCND1, CDKN1A, CREB1, CXCL8, E2F1, ESR1, JAK1, MAP3K12, MEF2D, NCOA3, PAK5, PTEN, RB1, RBL2, S1PR1, STAT3, TGFBR2, TLR7, TNF, VIM	2.295
hsa-miR-185-5p	AKT1, CCNE1, CDC42, CDK6, RHOA	1.596
hsa-miR-191-5p	IL6, TLR3	2.58
hsa-miR-210-3p	FGFRL1	1.438
hsa-miR-210-3p	PTPN1	1.438
hsa-miR-3619-5p	BAX, PTEN	−1.478
hsa-miR-22-3p	ESR1, PPARA, SRF	1.403
hsa-miR-92a-3p	BCL2L11, BMPR2, CCNE2, CDKN1A, ITGA5, MAP2K4, PTEN	−1.498
**Exosomal miRNAs involved in mTOR signaling**		
hsa-let-7b-5p	EIF3J, EIF4G2, HMOX1, HRAS, KRAS, NRAS, Ras, RHOB, RHOG	−1.712
hsa-miR-99b-5p	FGFR3, MTOR, RPTOR	1.364
hsa-miR-124-3p	PGF, PRKD1, RHOG	1.404
hsa-miR-5189-5p	AKT2	1.782
hsa-miR-138-5p	RHOC	1.561
hsa-miR-5195-3p	EIF4E, IRS1	1.505
hsa-miR-152-3p	RPS6KA5	−1.353
hsa-miR-155-5p	PRKCI, RHEB, RHOA	1.722
hsa-miR-16-5p	EIF4E, FGFR1, GRB2, HMOX1, MAPK3, PPP2R5C, RHOT1, VEGFA	2.44
hsa-miR-20a-5p	VEGFA	2.295
hsa-miR-181c-5p	KRAS	−1.312
hsa-miR-185-5p	AKT1, RHOA	1.596
hsa-miR-222-3p	DDIT4, DIRAS3, PIK3R1, PPP2R2A	1.363
hsa-miR-31-5p	HIF1A, PPP2R2A	1.494
hsa-miR-494-3p	HMOX1	1.464
**Exosomal miRNAs involved in Autophagy**		
hsa-let-7b-5p	VPS39	−1.712
hsa-miR-99b-5p	MTOR	1.364
hsa-miR-5195-3p	LAMP2	1.505
hsa-miR-155-5p	ATG3	1.722
hsa-miR-16-5p	ATG9A., BCL2, SQSTM1	2.44
hsa-miR-20a-5p	BCL2	2.295
hsa-miR-181c-5p	BCL2	−1.312

## Data Availability

The data set will be available upon request from the authors.
